# Mineralocorticoid axis activity and cardiac remodeling in patients with ACTH-dependent Cushing’s syndrome

**DOI:** 10.1530/EC-24-0617

**Published:** 2025-01-27

**Authors:** Peter Wolf, Simon Travers, Oliver Domenig, Stephanie Baron, Anne Blanchard, Khaoula Bouazizi, Nadjia Kachenoura, Sylvie Salenave, Marko Poglitsch, Alban Redheuil, Severine Trabado, Jacques Young, Philippe Chanson, Peter Kamenický

**Affiliations:** ^1^ Université Paris-Saclay, Inserm, Physiologie et Physiopathologie Endocriniennes, Assistance Publique-Hôpitaux de Paris, Hôpital Bicêtre, Service d’Endocrinologie et des Maladies de la Reproduction, Centre de Référence des Maladies Rares de l’Hypophyse, Le Kremlin-Bicêtre, France; ^2^ Medical University of Vienna, Department of Internal Medicine III, Division of Endocrinology and Metabolism, Vienna, Austria; ^3^ Université Paris Cité, PARCC, Inserm, Paris, France; ^4^ Assistance Publique-Hôpitaux de Paris, Hôpital Européen Georges Pompidou, Service de Physiologie, Paris, France; ^5^ Université Paris-Saclay, Inserm, UMR-S 1180, Orsay, France; ^6^Assistance Publique-Hôpitaux de Paris, Hôpital Européen Georges Pompidou, Laboratoire de Biochimie, Paris, France; ^7^ Attoquant Diagnostics, Vienna, Austria; ^8^ Sorbonne Université, CNRS, Inserm, Laboratoire d’Imagerie Biomédicale, LIB, Paris, France; ^9^ Institut de Cardiométabolisme et Nutrition (ICAN), Paris, France; ^10^ Assistance Publique-Hôpitaux de Paris, Hôpital Pitié-Salpêtrière, Unité d’Imagerie Cardiovasculaire et Thoracique, APHP.SU, Paris, France; ^11^ Assistance Publique-Hôpitaux de Paris, Hôpital Bicêtre, Service de Génétique Moléculaire, Pharmacogénétique et Hormonologie, Le Kremlin-Bicêtre, France

**Keywords:** Cushing’s syndrome, hypertension, angiotensin, aldosterone, RAS system

## Abstract

**Background:**

Arterial hypertension and left ventricular hypertrophy and remodeling are independent cardiovascular risk factors in patients with Cushing’s syndrome. Changes in the renin–angiotensin system and in the mineralocorticoid axis activity could be involved as potential mechanisms in their pathogenesis, in addition to cortisol excess.

**Methods:**

In this ancillary study of our previous study prospectively investigating patients with ACTH-dependent Cushing’s syndrome by cardiac magnetic resonance imaging (NCT02202902), 11 patients without any interfering medication were cross-sectionally compared to 20 control subjects matched for age, sex and body mass index. Angiotensin metabolites and adrenal steroids were measured by liquid chromatography tandem mass spectrometry, and their relation to blood pressure and cardiac structure was evaluated.

**Results:**

Concentrations of angiotensin I and angiotensin II were comparable, but the angiotensin-converting enzyme activity was significantly lower (2.19 (1.67; 3.08) vs 4.07 (3.1; 5.6); *P* < 0.001) in patients compared to controls. Aldosterone concentrations were significantly lower (6.9 (6.9; 124.1) vs 239.9 (181.4; 321.9) pmol/L; *P* < 0.001) in the group of patients, but adrenal aldosterone precursor metabolites were comparable between patients and controls. Inverse correlations were observed for 24 h urinary free cortisol and aldosterone with the ratio of left ventricular mass to end-diastolic volume (*r* = 0.470, *P* = 0.012 and *r* = −0.367, *P* = 0.046, respectively).

**Conclusions:**

We describe a disease-specific profile of angiotensin metabolites in patients with ACTH-dependent Cushing’s syndrome. Low levels of aldosterone in the presence of unchanged precursor metabolites indicate a direct inhibitory action of cortisol excess on the aldosterone synthase. Furthermore, glucocorticoid excess *per se* drives cardiac muscle remodeling.

## Background

Cushing’s syndrome is a severe disease resulting from endogenous hypercortisolism or from iatrogenic overexposure to pharmacological doses of exogenous corticosteroids (1). Morbidity and mortality are markedly increased in patients with Cushing’s syndrome, mainly because of cardiovascular diseases (2, 3).

Hypertension is among the most prevalent cardiovascular risk factors and can be observed in about 80% of patients with chronic hypercortisolism (4). Nevertheless, the pathophysiology of hypertension in Cushing’s syndrome is complex and only partially understood. Underlying mechanisms include the stimulation of the mineralocorticoid receptor by high cortisol concentrations, an increased sensitivity to sympathetic stimuli and altered concentrations of other vasoregulatory mediators (5).

In addition, changes in the renin–angiotensin system (RAS) may be involved. The RAS consists of an enzymatic cascade and plays a central role in water and electrolyte homeostasis and blood pressure (BP) regulation (6). Angiotensin II, generated from angiotensin I following the cleavage of angiotensinogen, stimulates aldosterone synthesis, but also directly promotes vasoconstriction and inflammation (7). On the other hand, an alternative angiotensin-converting enzyme (ACE)-independent RAS pathway might counteract adverse actions of angiotensin II (8, 9). Previously described alterations of the RAS in patients with Cushing’s syndrome include higher levels of angiotensinogen (10), despite lower (10, 11) or unchanged (12, 13) renin levels and an increased sensitivity to angiotensin II (12, 14). However, the results of these studies are limited by an old methodology (11, 12, 13), a heterogenous cohort of patients, including patients with ACTH-independent adrenal causes of hypercortisolism (11, 12) or the inclusion of patients treated by drugs well known to interfere with the RAS (10).

Furthermore, besides hypercortisolism *per se*, alterations in mineralocorticoid activity might explain changes in cardiac structure and function observed in patients with Cushing’s syndrome (15, 16), given the well-known adverse effects of angiotensin II and aldosterone on the heart (7, 17).

Therefore, the aim of the present study was to evaluate mineralocorticoid axis activity and angiotensin metabolites in a well-phenotyped cohort of patients with mild-to-moderate ACTH-dependent Cushing’s syndrome using state-of-the-art methodology using mass spectrometry. The relation of glucocorticoid and mineralocorticoid axis activity on the heart was investigated by cardiac magnetic resonance imaging.

## Methods

### Study design

We performed an ancillary study on a subset of patients from our previous prospective cross-sectional study in patients with endogenous Cushing’s syndrome (16). The study was approved by the local Ethics Committee (Comité de Protection des Personnes Ile de France VII, Approval No. 11-034), and all subjects gave written informed consent to participate in the study. The study protocol was registered at clinicaltrials.gov (NCT02202902).

### Subjects

Study participants were recruited from June 2014 to September 2018 and were consecutively enrolled. Eligible patients were those with an ACTH-dependent Cushing’s syndrome aged 18 to 70-year with 24-h urinary-free cortisol (UFC) excretion above the upper limit of normal at inclusion visit. Diagnosis of Cushing’s syndrome was based on the criteria recommended by clinical practice guidelines, including elevated UFC excretion, lack of cortisol suppression during the overnight 1 mg dexamethasone suppression test and the loss of circadian rhythm with elevated midnight cortisol levels. ACTH dependency was defined by an ACTH >20 pg/mL (1, 2). Both treatment-naive patients and patients uncured by previous transsphenoidal surgery could participate in the study. Women were eligible if they did not receive estrogen-based oral contraception for more than three months before inclusion.

Eligible control subjects were asymptomatic individuals matched for age, sex and body mass index (BMI) without a history of cardiovascular disease and cardiovascular risk factors, including hypertension, diabetes mellitus and dyslipidemia.

Exclusion criterion for all subjects was the routine intake of one of the following drug classes potentially interfering with the renin–angiotensin system (RAS) at the time of study entry: ACE inhibitors, angiotensin type 2 (AT2) receptor antagonists, spironolactone, thiazide diuretics and beta blockers (18). Other exclusion criteria were pregnancy, breastfeeding, acute infectious disease within the last seven days before inclusion, drug allergy and contraindication to magnetic resonance imaging.

### Interventions

Patients were investigated before treatment and were compared to a group of control subjects. Treatment of Cushing’s syndrome was performed independently of study-related activities according to routine clinical practice recommendations (1, 2). A subgroup of four patients was reinvestigated after clinically and biochemically confirmed disease remission.

Study participants underwent a detailed clinical and biochemical examination as previously reported (15). Anthropometric characteristics such as body weight, BMI, waist circumference and BP were assessed systematically. Blood was drawn in the morning after an overnight fast to measure concentrations of glucose, insulin, HbA1c, total cholesterol, HDL-cholesterol, LDL-cholesterol, triglycerides and electrolytes and parameters of liver and kidney function.

Hypertension was diagnosed by a BP of >140/90 mmHg, and diabetes mellitus was diagnosed by a fasting blood glucose of >7.0 mmol/L, a random glucose measurement >11.0 mmol/L or a HbA1c > 6.5%, both defined according to the World Health Organization criteria.

### Laboratory parameters

Laboratory parameters were assessed by using standard analytical methods as previously reported (15). Serum cortisol concentrations were measured with a solid-phase chemiluminescent competitive immunoassay using a polyclonal antibody (Siemens Healthcare Diagnostics Products Llanberis, UK). UFC excretion in patients was reported as the average of three consecutive daily 24 h urine samples and was determined on diurnal urine samples collected without the use of preservatives, after dichloromethane extraction by a solid-phase competitive chemiluminescent enzyme immunoassay (Siemens Healthcare Diagnostics Products Llanberis, UK). HOMA-IR as an estimate of insulin sensitivity was calculated as previously reported (19).

### RAS fingerprint

Equilibrium concentrations of angiotensin I, angiotensin II, angiotensin III, angiotensin IV, angiotensins 1–7 and angiotensins 1–5 and of the steroid aldosterone were measured following *ex vivo* equilibration and subsequent stabilization of conditioned plasma samples as described previously (8, 20, 21). Samples were spiked with stable isotope-labeled internal standards for individual angiotensin (200 pg) and deuterated (D4) aldosterone (500 pg). Following C18-based solid-phase extraction, samples were subjected to liquid chromatography–tandem mass spectrometry (LC–MS/MS) analysis using a reversed-phase analytical column (Acquity UPLC® C18, Waters) operating in line with a XEVO TQ-S triple quadrupole mass spectrometer (Waters Xevo TQ/S, USA) in multiple reaction monitoring mode. Internal standards were used to correct for analyte recovery across the sample preparation procedure in each individual sample. Analyte concentrations were calculated from integrated chromatograms considering the corresponding response factors determined in appropriate calibration curves in serum matrix, on condition that integrated signals exceeded a signal-to-noise ratio of 10. Equilibrium levels of angiotensin I and angiotensin II and aldosterone levels were used in RAAS-Triple-A evaluation, involving the calculation of angiotensin-based markers for plasma renin activity (PRA-S: angiotensin I + angiotensin II), angiotensin-converting enzyme (ACE-S: angiotensin II/angiotensin I) and the AA2-Ratio (aldosterone/angiotensin II), as a marker for adrenal responsiveness to angiotensin II signaling in releasing aldosterone.

### Adrenal steroid metabolomics

#### Materials

Isolute SLE+ 400 μL (Biotage®, Sweden) 96-well solid–liquid extraction plates were used for sample preparation. Commercially available steroids (aldosterone, 18-hydroxycorticosterone, cortisol, cortisone, 21-deoxycortisol, 11-deoxycortisol, 11-deoxycorticosterone, 17-hydroxyprogesterone, delta-4-androstenedione and corticosterone) and their corresponding internal standards (cortisol-D4, cortisone-D7, 21-desoxycortisol-D8, 11-desoxycortisol-D5, corticosterone-D8, aldosterone-D8, 17-hydroxyprogesterone-D8, 11-deoxycorticosterone-C13 and delta-4-androstenedione-C13) were purchased from Cerilliant® (USA). 18-hydroxycortisol was purchased from Steraloids® (USA). Polypropylene 1 mL 96-well collection plates and Cap-mat 7 mm round plugs were purchased from Waters® (UK). LC–MS-grade methanol was purchased from LiChrosolv® (Merck®, USA) for use as the mobile phase. For sample preparation, methanol and LC–MS-grade acetonitrile were purchased from Biosolve® (France). LC–MS-grade water was produced on-site. Commercial steroid free serum (MP Biomedicals®, USA) was spiked with known concentrations of previously listed steroids in methanol or acetonitrile solution for calibrating samples and quality controls preparation.

### Sample preparation

250 μL sample were mixed with 50 of internal standards before distributing 275 μL on SLE+ 96-well plate (Biotage®, Sweden) for extraction. After steroid elution with dichloromethane (Acros Organics®, France), eluate was evaporated under azote and then reconstituted with 70 μL mobile phase consisting of 45% methanol and 55% ammonium formate 5 mM.

### Ultra-performance liquid chromatography–tandem mass spectrometry method

Chromatography was performed on a Waters® Acquity TM UPLC I-Class System (UK) with a CORTECS® UPLC C18 2.1 × 100 mm 1.6 μm column maintained at 50 °C (Waters®). 25 μL reconstituted sample were injected into the system with an ammonium formate 5 mM (A) and methanol (B) mobile phase (55:45 v/v initial conditions). Over 10 min methanol gradient went from 45 to 100% at 0.35 mL/min. Then, 100% B was maintained for 1 min before 1.50 min of column re-equilibration to initial conditions before next injection. Eluate from the column was injected into a Waters Xevo® TQ-S tandem mass spectrometer operating in the positive-electrospray ionization mode. In the MRM mode, two transitions were recorded for each measured steroid. Data were analyzed with MassLynx® 4.1 SCN905 software (Waters®).

### Cardiac magnetic resonance imaging

Cardiac magnetic resonance imaging measurements were performed at baseline and after treatment in a tertiary academic reference hospital, as previously described (15, 16). Images were obtained from a 3T magnet (Prisma, Siemens, Germany). Cine images were acquired using a balanced steady-state-free precession sequence in a stack of short-axis slices encompassing the left ventricle. Such images were then used to estimate both left ventricular mass and its end-diastolic and end-systolic volumes using a dedicated software (QMass, Medis, The Netherlands).

### Statistical analysis

No sample size calculation was performed, and all eligible patients without interfering medication were included. Quantitative variables are expressed as median (Q1;Q3). Qualitative variables are expressed as frequency (percent). Differences between controls and patients with Cushing’s syndrome were analyzed with the Mann–Whitney U test for quantitative variables and with the Chi-squared test for qualitative variables. The effects of Cushing’s syndrome treatment were analyzed with paired *t*-tests. Associations between quantitative outcomes were analyzed using Spearman correlation. Multiple regression analysis was performed to identify predictors of left ventricular mass/end-diastolic volume. Parameters with *P* value <0.2 in the univariate correlation analysis were included. *P* values below 0.05 were considered to denote statistical significance.

## Results

### Patients’ characteristics

Eleven patients with active ACTH-dependent Cushing’s syndrome and 20 control subjects were investigated. Ten patients had Cushing’s disease and one patient had ectopic ACTH secretion due to breast cancer. Two patients with Cushing’s disease were included following disease recurrence after initial pituitary surgery, and all other patients were newly diagnosed. In the absence of severe complications related to hypercortisolism (22), all patients can be considered as having a mild-to-moderate disease activity. One patient was substituted with levothyroxine, and pituitary insufficiency was not present in other patients.

The presence of diabetes was higher in patients compared to controls. Systolic and diastolic BP was not different. Potassium levels were lower in the group of patients compared to the control group.

Cardiac magnetic resonance imaging showed a reduction in left ventricular diastolic volume and stroke volume in patients compared to controls. Left ventricular mass was not different, but the ratio of left ventricular mass/end-diastolic volume, a surrogate for ventricular remodeling, was increased in patients compared to controls. A detailed description of clinical and biochemical characteristics is given in Table 1 and in Supplemental Table S1 (see section on Supplementary materials given at the end of the article).

**Table 1:** Clinical and biochemical characteristics of patients with active ACTH-dependent Cushing’s syndrome and matched controls.

	Patients with Cushing’s syndrome	Control group	*P* value (between group differences)
	*n* = 11	*n* = 20	
**Clinical parameters**			
Age (years)	32 (26; 36)	39 (32; 46)	0.72
Sex (female%)	10 (91)	16 (80)	0.63
Body mass index (kg/m^2^)	33 (20; 34)	29 (24; 35)	0.86
Presence of arterial hypertension (*n*%)	3 (27)	3 (15)	0.64
Presence of diabetes (*n*%)	5 (45)	0 (0)	0.003
Systolic blood pressure (mmHg)	125 (118; 145)	119 (113; 136)	0.28
Diastolic blood pressure (mmHg)	80 (67; 85)	72 (66; 77)	0.09
**Biochemical parameters**			
24 h urinary-free cortisol (μg/24)	366 (230; 869)	26 (18; 34)	0.001
Potassium (mmol/L)	4.2 (4; 4.5)	4.45 (4.3; 4.6)	0.044
Fasting glucose (mmol/L)	5.1 (4.5; 5.5)	4.9 (4.5; 5.2)	0.77
HOMA-IR	1.87 (1.2; 4.2)	1.61 (1.0; 3.3)	0.53
HbA1c (%)	5.9 (5.2; 6.6)	5.3 (5.1; 5.5)	0.09
**Cardiac magnetic resonance imaging parameters**			
Left ventricular end-systolic volume index (mL/m^2^)	29.7 (26.8; 31.9)	33.1 (28.1; 36.3)	0.21
Left ventricular end-diastolic volume index (mL/m^2^)	69.5 (65.4; 79.9)	83.1 (74.1; 91.8)	0.015
Left ventricular stroke volume index (mL/m^2^)	42.2 (36.6; 49.5)	50.3 (47.1; 55.4)	0.007
Left ventricular ejection fraction (%)	59.2 (53.3; 63.0)	61.1 (58.6; 62.6)	0.45
Left ventricular mass index (g/m^2^)	52.2 (45.5; 57.7)	51.9 (44.9; 55.8)	0.78
Left ventricular mass/end-diastolic volume (g/mL)	0.73 (0.63; 0.78)	0.60 (0.56; 0.70)	0.049

In the subgroup of patients re-examined after successful treatment, two patients were cured by pituitary surgery and two patients were treated by ketoconazole, following persistent disease after initial pituitary surgery. UFC was normalized in all patients, and no patient was hypertensive at follow-up.

### Angiotensin peptide profiles and their relation to aldosterone

**Figure 1:**
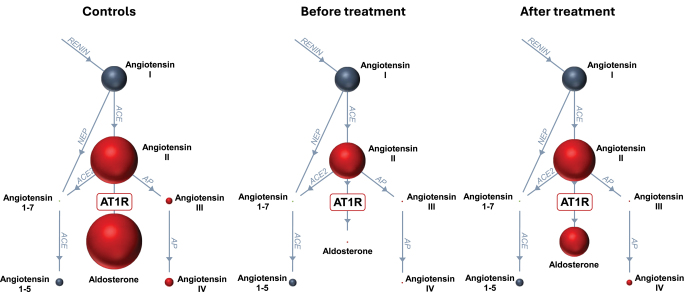
Angiotensin metabolite profiles in the control group (left column) and patients before (central column) and after (right column) biochemical disease control of Cushing’s syndrome. The sizes of spheres represent absolute concentrations of angiotensin metabolites (pmol/L, median value) analyzed by mass spectrometry; ACE, angiotensin-converting enzyme; Aldo, aldosterone; AP, aminopeptidase; AT1R, angiotensin 2 type 1 receptor; NEP, neutral endopeptidase.

Angiotensin metabolites were compared between the group of patients with Cushing’s syndrome and controls. All results are reported in detail in Table 2, and an illustration of angiotensin metabolites in patients before and after treatment and in controls is shown in Fig. 1. With regard to the classical RAS pathway, angiotensin I levels were comparable between patients and controls. Concentrations of angiotensin II were about 40% lower in the group of patients. This reflects a significantly reduced ACE activity in Cushing’s syndrome patients compared to controls. No difference in angiotensin metabolites in the alternative, ACE-independent RAS pathway was observed. Aldosterone concentrations and the AA2 ratio were significantly lower in patients compared to controls.

**Table 2:** Angiotensin metabolites in patients with Cushing’s syndrome and controls.

	Patients with Cushing’s syndrome	Control group	*P* value (between group differences)
	*n* = 11	*n* = 20	
**Classical RAS pathway**			
Ang I (pmol/L)	49.1 (27.9; 74.8)	50.3 (18.7; 78.9)	0.76
Ang II (pmol/L)	101.2 (43.5; 206.2)	173.8 (91.7; 330.9)	0.23
Ang III (pmol/L)	1.3 (1.3; 4.3)	3.17 (1.3; 5.7)	0.36
Ang IV (pmol/L)	1.0 (1.0; 5.9)	5.52 (2.9; 8.3)	0.09
Aldosterone (pmol/L)	6.9 (6.9; 124.1)	239.9 (181.4; 321.9)	<0.001
PRA-S (pmol/L)	129.1 (92.6; 296.9)	237.1 (117.9; 409.9)	0.38
AA2 ratio	0.38 (0.14; 1.12)	1.23 (0.8; 2.54)	0.003
ACE-S	2.19 (1.67; 3.08)	4.07 (3.1; 5.6)	<0.001
**Alternative RAS pathway**			
Ang 1–7 (pmol/L)	1.5 (1.5; 6.3)	1.5 (1.5; 1.5)	0.47
Ang 1–5 (pmol/L)	4.25 (2.9; 11.5)	4.38 (2.4; 6.4)	0.45

Ang, angiotensin; PRA-S, plasma renin activity surrogate; AA2 ratio, aldosterone/angiotensin II ratio; ACE-S, angiotensin-converting enzyme surrogate.

In the subgroup of patients who were investigated before and after treatment, biochemical disease remission resulted in a trend toward normalization of the angiotensin profile comparable to the control group. The results of the subgroup of patients before and after treatment are reported in detail in Supplemental Table 1.

### Adrenal steroid metabolomics analysis

**Figure 2:**
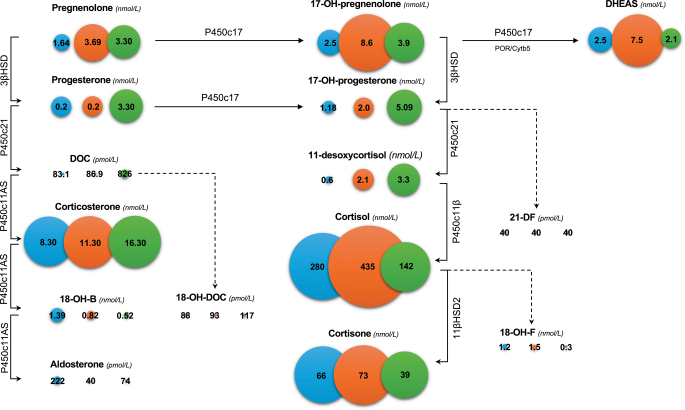
Steroidomic profiles of control group (blue circles), patients with Cushing’s syndrome before treatment (orange circles) and after treatment (green circle). Data are given as means; the sizes of spheres estimate the absolute concentrations.

Data of adrenal steroid metabolites in patients and controls are shown in detail in Table 3 and are illustrated in Fig. 2. Pregnenolone concentrations were higher in the group of patients with Cushing’s syndrome. Besides significantly lower aldosterone concentrations, aldosterone precursor metabolites were comparable between the two groups except for 18-OH-corticosterone (18-OH-B), which was lower in patients compared to controls. Precursor metabolites of the cortisol pathway were higher in patients with Cushing’s syndrome, but only differences in 17-OH-pregnenolone and 11-desoxycortisol were statistically significant. DHEAS levels were markedly higher in patients compared to controls. Aldosterone concentrations measured by both methodologies strongly correlated with each other (Supplemental Fig. 1).

**Table 3:** Adrenal steroid profiling in patients with Cushing’s syndrome and controls (median (min; max)).

	Patients with Cushing’s syndrome	Control group	*P* value (between group differences)
	*n* = 11	*n* = 20	
Pregnenolone (nmol/L)	3.69 (1.8; 8.5)	1.64 (1; 2.4)	0.009
Progesterone (nmol/L)	0.2 (0.2; 0.35)	0.2 (0.2; 3.2)	0.73
**Aldosterone pathway**			
DOC (pmol/L)	86.9 (53.2; 180.4)	83.1 (40.2; 131.9)	0.58
Corticosterone (nmol/L)	11.3 (5.5; 26.3)	8.3 (4.1; 13.0)	0.23
18-OH-corticosterone (nmol/L)	0.82 (0.6; 1.4)	1.39 (1.1; 1.6)	0.049
18-OH-DOC (pmol/L)	93.2 (40; 309.6)	87.8 (53.3; 189.6)	0.95
Aldosterone (pmol/L)	40 (40; 135.51)	222 (161; 335)	<0.001
**Cortisol pathway**			
17-OH-Pregnenolone (nmol/L)	8.6 (3.9; 12.9)	2.5 (1.5; 3.9)	<0.001
17-OH-progesterone (nmol/L)	2 (1.4; 3.2)	1.18 (0.6; 2.7)	0.28
11-desoxycortisol (nmol/L)	2.1 (0.8; 3.5)	0.6 (0.4; 0.9)	0.008
21-DF (pmol/L)	40 (40; 50.9)	40 (40; 60.5)	0.67
Cortisol (nmol/L)	435 (323; 601)	280 (214; 366)	0.16
Cortisone (nmol/L)	73.4 (54.8; 88.6)	65.6 (50.9; 79.2)	0.58
18-OH-F (nmol/L)	1.5 (0.3; 3)	1.2 (0.9; 2)	0.77
**Androgen pathway**			
DHEAS (nmol/L)	7.5 (5.5; 11.6)	2.5 (1.9; 3.2)	<0.001

DOC, deoxycortisol; 18-OH-DOC, 18-OH-deoxycorticosterone; 21-DF, 21-deoxycortisol; 18-OH-F, 18-OH-corticostosterone; DHEAS, dehydroepiandrosterone.

Data of the subgroup of patients who were investigated before and after successful treatment are reported in Supplemental Table 1. No significant differences were observed.

### Cortisol and aldosterone as determinants of cardiac remodeling

**Figure 3:**
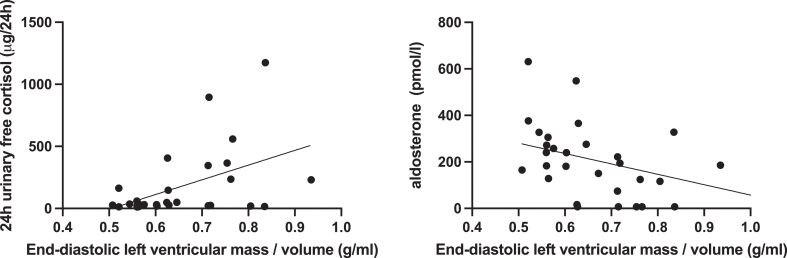
Univariate correlation analysis of left ventricular mass/end-diastolic volume ratio and 24 h urinary free cortisol (left) and aldosterone (right) in the whole cohort.

The results of univariate correlation analysis are reported in Table 4. The correlation between 24 h UFC or aldosterone with left ventricular mass/end-diastolic volume is shown in Fig. 3. 24 h UFC excretion positively correlated with systolic and diastolic BP, whereas aldosterone concentration negatively correlated with diastolic BP. Levels of 24 h UFC, 17-OH-P, 11-desoxycortisol and DHEAS positively correlated with left ventricular mass/end-diastolic volume. Inversely, negative associations between left ventricular mass/end-diastolic volume and aldosterone and 18-OH-B were observed. Aldosterone, the AA2 ratio and 18-OH-B positively correlated with end-diastolic volume.

**Table 4:** Univariate correlation analysis of selected clinical parameters and parameters of cardiac structure and function with angiotensin metabolites and adrenal steroids.

	24h UFC	11-DOC	DHEAS	18-OH-B	Aldo	Ang II	A/AT2
Systolic blood pressure	*0.412*; *P* = *0.026*	0.273; *P* = 0.14	0.223; *P* = 0.24	−0.283; *P* = 0.12	−0.332; *P* = 0.7	−0.231; *P* = 0.21	0.144; *P* = 0.44
Diastolic blood pressure	*0.454*; *P* = *0.013*	0.062; *P* = 0.74	*0.274*; *P = 0.14*	*−0.421*; *P* = *0.018*	*−0.406*; *P* = *0.023*	−0.160; *P* = 0.39	−0.105; 0.58
Sodium	0.118; *P* = 0.54	−0.016; *P* = 0.93	0.128; *P* = 0.50	−0.046; *P* = 0.81	−0.127; *P* = 0.49	−0.065; *P* = 0.73	0.101; *P* = 0.58
Potassium	−0.244; *P* = 0.20	−0.237; *P* = 0.20	*−0.398*; *P* = *0.029*	−0.116; *P* = 0.54	0.221; *P* = 0.23	0.136; *P* = 0.47	0.109; *P* = 0.56
ESV	−0.308; *P* = 0.11	−0.224; *P* = 0.23	−0.266; *P* = 0.16	*0.561*; *P* = *0.001*	*0.550*; *P* = *0.002*	−0.056; *P* = 0.77	0.094; *P* = 0.62
EDV	−0.275; *P* = 0.16	−*0.429*; *P* = *0.018*	*−0.375*; *P* = *0.045*	*0.527*; *P* = *0.003*	*0.405*; *P* = *0.026*	−0.128; *P* = 0.50	0.297; *P* = 0.11
SV	−0.165; *P* = 0.40	*−0.480*; *P* = *0.007*	*−0.371*; *P* = *0.048*	0.333; *P* = 0.07	0.150; *P* = 0.43	−0.154; *P* = 0.42	*0.387*; *P* = *0.035*
EF	0.214; *P* = 0.21	−0.144; *P* = 0.45	−0.062; *P* = 0.75	*−0.284*; *P* = *0.13*	*−0.373*; *P* = *0.043*	−0.004; *P* = 0.98	0.197; 0.29
LVM	0.240; *P* = 0.22	0.141; *P* = 0.46	−0.56; *P* = 0.77	−0.067; *P* = 0.72	−0.090; *P* = 0.64	−0.297; *P* = 0.11	0.116; *P* = 0.54
LVM/EDV	*0.470*; *P* = *0.012*	*0.522*; *P* = *0.003*	0.257; *P* = 0.178	*−0.461*; *P* = *0.010*	*−0.367*; *P* = *0.046*	−0.075; *P* = 0.69	−0.151; 0.43

24h UFC, 24 h urinary free cortisol; 11-DOC, 11-desoxycortisol; 18-OH-B, 18-OH-corticosterone; Aldo, aldosterone; Ang II, angiotensin 2; A/AT2, aldosterone/angiotensin 2 ratio; ESV, left ventricular end-systolic volume; EDV, left ventricular end-diastolic volume; SV, left ventricular stroke volume; EF, left ventricular ejection fraction; LVM, left ventricular mass; LVM/EDV, left ventricular mass/end-diastolic volume; *P* values <0.05 are italicized.

No parameter predictive for left ventricular mass/end-diastolic volume was identified in multiple regression analysis.

## Discussion

In the present study, we used liquid chromatography–tandem mass spectrometry techniques in combination with angiotensin peptide equilibrium analysis to characterize mineralocorticoid axis activity in patients with ACTH-dependent Cushing’s syndrome compared to matched healthy subjects. We found a specific RAS profile in patients with ACTH-dependent Cushing’s syndrome with concentrations of angiotensins I and II comparable to controls, but a moderately reduced ACE activity. Suppressed aldosterone concentrations in the presence of unchanged precursor metabolites indicate a direct inhibitory effect on the aldosterone synthase in a state of hypercortisolism. We further showed that patient with Cushing’s syndrome had an increased left ventricular mass/end-diastolic volume ratio indicative of concentric ventricular remodeling related to cortisol excess.

Changes in the RAS are considered as important contributors to the development of arterial hypertension in Cushing’s syndrome (5). Previous studies in patients have reported increased concentrations of angiotensinogen, the substrate for renin, which is then further cleaved to angiotensin I (see Fig. 1) (10). In line with these findings, animal models show stimulated angiotensinogen expression following dexamethasone exposure in the isolated rat liver (23). However, based on our data, this previously prescribed increase in angiotensinogen does not result in a general upregulation of the RAS since circulating concentrations of angiotensins I and II were comparable between patients and controls. ACE activity, estimated by calculating the ratio of angiotensins I and II, was even significantly reduced in the patient group. On the other hand, evidence suggests an increased angiotensin II receptor expression, which has been demonstrated in smooth muscle cells (24) and peripheral blood cells (13) following glucocorticoid exposure *in vitro*. Moreover, the increase in diastolic BP following an infusion with angiotensin II was substantially higher in patients with Cushing’s syndrome due to an adrenal adenoma compared to a healthy control group (12). We therefore suppose that not a general increase in RAS activity but an increased susceptibility to angiotensin II might contribute to the pathophysiology of arterial hypertension in Cushing’s syndrome. The reduced ACE activity might result from negative feedback by increased angiotensin II receptor activation or mineralocorticoid receptor stimulation in a state of hypercortisolism. No differences in angiotensin metabolites of alternative RAS pathways could be found.

In line with previous reports (10, 25, 26), aldosterone concentrations were significantly lower in patients with ACTH-dependent Cushing’s syndrome compared to controls. Aldosterone levels below the lower limit of detection were observed in 7 out of 12 (58%) patients. Interestingly, precursors of aldosterone synthesis were normal in steroid metabolomics analysis, demonstrating a direct inhibition of the aldosterone synthase by cortisol excess. Furthermore, the stimulatory effect of ACTH on aldosterone secretion under physiological conditions (27, 28) might not be of relevance in patients with overt hypercortisolism.

In four patients reassessed following biochemical disease control, aldosterone trended to increase but was still markedly lower (*P* < 0.001) compared to the control group after a mean follow-up of 12 ± 10 months (Supplemental Table S1), suggesting that the restauration of the typical mineralocorticoid axis activity might be a long process. Of note, two of these patients were on ketoconazole, and only two were cured by surgery. Given the well-known action of ketoconazole on multiple steroidogenic enzymes, the adrenal steroid metabolomic profile at follow-up must be interpreted with caution.

Systolic and diastolic BP positively correlated with the extent of hypercortisolism estimated by UFC excretion, whereas diastolic BP was negatively associated with aldosterone concentration. Potassium levels were lower in patients compared to the control group. This is due to the direct mineralocorticoid action of cortisol. Whereas cortisol binding to the mineralocorticoid receptor is immediately inactivated to cortisone by the 11beta-hydroxysteroid dehydrogenase type 2 (11b-HSD2) under physiological conditions, this enzyme is saturated in a hypercortisolic state (29, 30). This results in mineralocorticoid receptor hyperactivation by cortisol despite low levels of circulating aldosterone.

While the design of our study does not allow to draw conclusions regarding the optimal antihypertensive treatment for patients with Cushing’s syndrome, the reduction in ACE activity does not support the use of an ACE inhibitor as first-line treatment as suggested by some authors (5, 31).

Evaluation of the heart structure and function by cardiac magnetic resonance imaging showed a smaller end-diastolic ventricular volume, a smaller stroke volume and an increased ratio of end-diastolic left ventricular mass/volume in patients included in the present study as compared to the control group, which was expected according to our previous publications (15, 16). The increase in the ratio of end-diastolic mass/volume reflects a concentric cardiac remodeling, which might be of special interest as it is a well-known contributor to adverse cardiovascular events, heart failure and sudden death (32). Interestingly, only 24 h UFC, precursor metabolites of cortisol synthesis and DHEAS positively correlated with the ratio of left ventricular mass/end-diastolic volume. Aldosterone concentrations correlated negatively with this parameter (see Fig. 3). This stands in contrast to observations in the general population where aldosterone is positively associated with myocardial hypertrophy (17). Of note, in contrast to other target tissues of mineralocorticoids, 11b-HSD2 is not expressed in cardiomyocytes (33), probably making the heart especially sensitive to an increase in cortisol exposure. In patients with Cushing’s syndrome, cortisol excess *per se* and its action on the mineralocorticoid receptor explain cardiac muscle changes.

In our study, arterial hypertension was present in only a minority of patients, which could be considered as a potential limitation. However, BP-lowering medication known to interfere with the RAS is considered as first-line antihypertensive treatment in patients with Cushing’s syndrome (34, 35). Excluding patients with concomitant RAS interfering treatment led to selection of patients with mild-to-moderate disease. On the other hand, applying these rigorous exclusion criteria allowed an in-depth characterization of circulating angiotensin metabolites and adrenal steroids for the first time in a well-selected cohort of patients. However, mineralocorticoid axis activity in severe hypercortisolism warrants further investigations. The prevalence of diabetes was significantly higher in patients compared to controls. However, in general population, obesity and insulin resistance are associated with an upregulation of the RAS (36).

Furthermore, it was not possible to measure plasma renin concentration in our study, as all samples were rapidly stored at −20° freezers during the study days, which might lead to cryoactivation and falsely elevated renin concentrations (37, 38). However, the plasma renin activity surrogate calculated from RAS fingerprint analysis was about 40% lower in the group of patients with Cushing’s syndrome compared to the control group, which is comparable to previous publications reporting renin concentrations below or within the normal range (10, 12).

In conclusion, patients with ACTH-dependent Cushing’s syndrome have concentrations of angiotensin I and angiotensin II within their typical ranges, but a moderately reduced ACE activity. Circulating aldosterone concentrations are suppressed without substantial changes in other precursor metabolites in the mineralocorticoid pathway, indicating a specific inhibitory action of cortisol excess on the aldosterone synthase. Cortisol excess *per se* is therefore responsible for the concentric left ventricular remodeling in patients with Cushing’s syndrome.

## Supplementary materials



## Declaration of interest

The authors declare that there is no conflict of interest that could be perceived as prejudicing the impartiality of the work.

## Funding

This work was supported by the Assistance Publique-Hôpitaux de Parishttps://doi.org/10.13039/501100009820 (PHRC AOR-12074).

## Author contribution statement

PW and PK conceived the original idea and designed the study. SB, PK, PC, SS and JY were responsible for subject’s recruitment. STrav, PW, OD, SB, PK, KB, NK, MP, AR and STrab were responsible for the performance of study-related experiments. PW, STrav, OD, MP and PK analyzed and interpreted the results of the study. PW, STrav and PK wrote the manuscript. All the authors reviewed and approved the manuscript.

## Data availability

All data generated or analyzed during this study are included in this published article or in the data repositories listed in the references. Source data are available upon request.
