# Supplementary material for: Mineralocorticoid axis activity and cardiac remodeling in patients with ACTH-dependent Cushing’s syndrome
Source: Endocr Connect. 2025 Jan 27;14(2):e240617. doi: 10.1530/EC-24-0617 (PMC11799754; doi:10.1530/EC-24-0617)
Supplement: Supplementary file 1 [file supplementary_materials.pdf]

Supplemental figure 1 – Correlation of aldosterone concentrations measured by RAS fingerprint and adrenal steroid metabolomics (r=0.945; p<0.001).

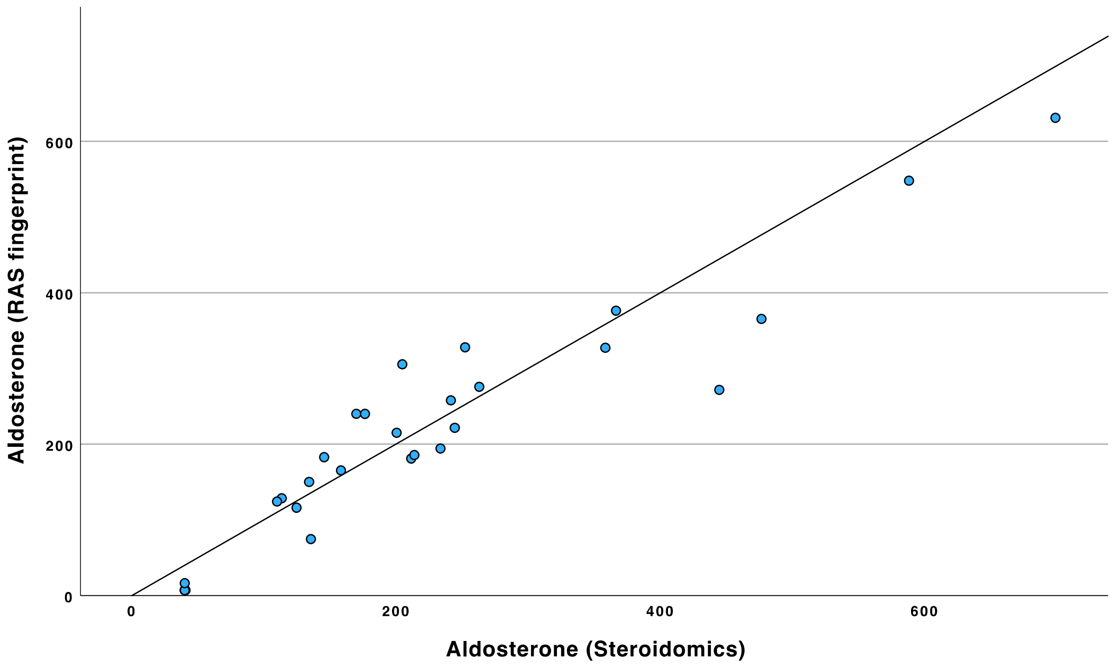

Supplemental table S1 – Additional biochemical characteristics in patients with active ACTH dependent Cushing's syndrome and matched controls

	**Patients with Cushing's syndrome**	**Control group**	**p-value**
**Biochemical parameters**			
Sodium (mmol/l)	142 (140; 142)	141 (140; 143)	0.64
Creatinine (mol/l)	67 (56; 75)	66 (61; 78)	0.89
Fasting insulin (mUI/l)	9 (6; 21)	10 (5; 17)	0.59
Triglycerides (mmol/l)	1.1 (0.8; 1.7)	1.1 (0.7; 1.4)	0.61
Total cholesterol (mmol/l)	5.0 (4.2; 5.4)	4.8 (4.5; 5.4)	0.83
LDL cholesterol (mmol/l)	2.8 (2.5; 3.1)	3 (2.4; 3.4)	0.47
HDL cholesterol (mmol/l)	1.4 (1.1; 1.8)	1.4 (1.1; 1.8)	1.0
ASAT (U/l)	24 (20; 36)	26 (22; 34)	0.52
ALAT (U/l)	32 (26; 45)	23 (18; 32)	0.09

Supplemental table S2 – Clinical and biochemical characteristics, concentrations of angiotensin metabolites and steroid metabolomic analysis in patients before and after biochemical control of Cushing's disease

	**Before treatment**	**After treatment**	**p-value**
	N=4	N=4	
**Clinical & biochemical parameters**			
**Body mass index** (kg/m^2)	27.4 (21; 33)	27.8 (21.8; 32.7)	0.91
**Systolic blood pressure** (mmHg)	115 (110; 123)	115 (109; 124)	0.91
**Diastolic blood pressure** (mmHg)	67 (62; 79)	64 (53; 69)	0.47
**24-hour urinary free cortisol** (µg/24)	284.5 (151; 773)	24.5 (12.5; 50.8)	0.12
**Potassium** (mmol/l)	4.3 (3.8; 4.6)	4.2 (3.9; 5.1)	0.49
**Sodium** (mmol/l)	141 (140; 143)	141 (140; 142)	0.39
**Creatinine** (mol/l)	72.5 (67.8; 75.8)	70.1 (65; 89)	0.66
**Angiotensin metabolites profile**			
**Classical RAS pathway**			
**Ang 1** (pmol/l)	41.1 (10.8; 69.6)	47.5 (4.0; 67.5)	0.848
**Ang 2** (pmol/l)	133.9 (34.1; 267.1)	138.8 (59.8; 425.9)	0.41
**Aldosterone** (pmol/l)	6.9 (6.9; 6.9)	69.4 (11.2; 156.8)	0.36
**PRA-S** (pmol/l)	174.9 (44.9; 336.8)	186.3 (74.7; 491.4)	0.46
**AA2 ratio**	0.66 (0.11; 1.9)	0.31 (0.18; 0.99)	0.60
**ACE-S**	3.35 (2.49; 3.91)	2.73 (4.84; 6.94)	0.13
**Alternative RAS pathway**			
**Ang 1-7** (pmol/l)	1.5 (1.5; 1.5)	1.5 (0.67; 1.5)	0.39
**Ang 1-5** (pmol/l)	4.9 (1; 10.8)	2.4 (1; 11.9)	0.72
**Ang 3** (pmol/l)	1.2 (1.3; 11.1)	1.3 (1.3; 11.7)	0.29
**Ang 4** (pmol/l)	1.7 (1; 8.9)	2.4 (1; 11.9)	0.36
**Adrenal steroid metabolomics profile**			
**Aldosterone pathway**			
**Pregnenolone** (nmol/l)	5.4 (2.5; 9.9)	3.3 (1; 8.1)	0.07
**Progesterone** (nmol/l)	0.28 (0.2; 1.2)	3.3 (1; 8.1)	0.27

**DOC** (pmol/l)	125.8 (73; 179)	826 (71; 1892)	0.22
**Corticosterone** (nmol/l)	15.9 (5.9; 25.9)	16.3 (1.4; 30.2)	0.99
**18-OH-B** (nmol/l)	1.14 (0.8; 2.5)	0.52 (0.3; 0.9)	0.19
**18-OH-DOC** (pmol/l)	191.3 (48.3; 315.1)	116.5 (43.8; 394.8)	1
**Aldosterone** (pmol/l)	40.3 (40; 40)	74 (40; 176.2)	0.42
**Cortisol pathway**			
**17-OH-Pregnenolone** (nmol/l)	11.3 (5.2; 13.9)	3.9 (1.0; 10.3)	0.23
**17-OH-Progesterone** (nmol/l)	2.04 (1.6; 3.5)	5.09 (1.4; 6.9)	0.21
**11-Desoxycortisol** (nmol/l)	2.18 (1.4; 3.2)	3.27 (0.3; 8.6)	0.55
**21-DF** (pmol/l)	45.5 (40; 50.9)	40 (40; 40)	0.18
**Cortisol** (nmol/l)	540 (425; 705)	142 (36; 315)	0.06
**Cortisone** (nmol/l)	84.9 (71.9; 97.1)	38.9 (11.2; 51.9)	*0.047*
**18-OH-F** (nmol/l)	2.7 (1.4; 5.0)	0.3 (0.3; 0.38)	0.07
**Androgen pathway**			
**DHEAS** (nmol/l)	10.8 (7.9; 10.8)	2.1 (0.8; 9.6)	0.37
